# Clinical and economic evaluation of robotic vs. freehand screw fixation in femoral neck fractures: advancing precision alignment in orthopedics

**DOI:** 10.3389/fsurg.2025.1699937

**Published:** 2025-12-16

**Authors:** Fawwaz Al-Smadi, Sajeda Al-Smadi, Xudong Xie, Yanzhi Zhao, Jiewen Liao, Mengfei Liu, Bobin Mi, Guohui Liu

**Affiliations:** 1 Department of Orthopedics, Union Hospital, Tongji Medical College, Huazhong University of Science and Technology, Wuhan, China; 2 Pediatric Health Nursing, Department of Allied Medical Sciences, Zarqa University College, Al-Balqa Applied University, Zarqa, Jordan

**Keywords:** femoral neck fractures, cannulated screw placement, robotic surgical procedures, surgical precision, cost-effectiveness

## Abstract

**Introduction:**

Femoral neck fractures are common and technically challenging injuries that often require internal fixation, with outcomes influenced by surgeon experience. This study compared clinical and economic outcomes of TiRobot-assisted vs. freehand cannulated screw fixation, focusing on alignment precision and anatomical considerations to assess the role of robotics in enhancing accuracy in orthopedic care.

**Methods:**

This single-center retrospective cohort study included 50 adult patients with femoral neck fractures treated between July 2023 and March 2024 at Wuhan Union Hospital. Patients were followed for 12 to 20 months and evenly divided into two groups: TiRobot-assisted (*n* = 25) and traditional freehand fixation (*n* = 25). Intraoperative parameters (operative time, blood loss, fluoroscopy frequency, and screw accuracy) and postoperative outcomes (hip function, complications, hospital stay, and surgical cost) were analyzed using appropriate statistical tests.

**Results:**

Operative time was significantly shorter in the TiRobot group (68 ± 14 min) than in the traditional group (81 ± 23.5 min; *p* = 0.028). Blood loss (27.68 ± 12.99 vs. 49.8 ± 15.03 mL; *p* < 0.001) and fluoroscopy use (12.24 ± 1.94 vs. 20.24 ± 2.47; *p* < 0.001) were significantly reduced. Screw parallelism was notably better in both anteroposterior (*p* = 0.008) and lateral views (*p* = 0.010). At the 12-month follow-up, Harris hip scores were higher in the TiRobot group (94 ± 8.5 vs. 87 ± 13.5; *p* = 0.014). Time to discharge (5.01 ± 1.46 vs. 7.03 ± 1.86 days; *p* < 0.001) and total hospital stay (7 ± 3 vs. 8 ± 3 days; *p* = 0.039) were shorter. Surgical cost was higher in the TiRobot group (CNY 28,998.31 ± 4,659.88 vs. CNY 17,653.63 ± 4,461.55; *p* < 0.001).

**Conclusions:**

TiRobot-assisted fixation offers greater precision, reduced intraoperative trauma, and faster recovery. Though more costly, its clinical advantages support its role in anatomically-precise orthopedic surgery.

## Introduction

Femoral neck fractures are the most prevalent form of hip fracture, making up over 50% of all hip injuries (1). Although individuals of any age group can suffer from these fractures, they predominantly affect the elderly population due to increased life expectancy (2, 3). According to relevant reports, the global incidence of hip fractures is rising steadily, currently resulting in disability for approximately 4.5 million people annually, with this number projected to escalate to 21 million within the next 40 years (4, 5). Consequently, the associated healthcare costs are also substantially increasing (6).

Surgical intervention is increasingly recommended for the majority of femoral neck fractures, including open or closed reduction and internal fixation, *in situ* fixation, total hip replacement, and hemiarthroplasty (1). Nonetheless, internal fixation is often complicated by femoral head avascular necrosis and non-union, which can significantly impair postoperative recovery and potentially necessitating secondary surgeries. Reducing these complications remains a significant challenge for surgeons and continues to be an active area of research (7).

Closed reduction with percutaneous hollow tension screw internal fixation is one of the primary surgical techniques utilized for the femoral neck fracture treatment. However, due to the complex anatomy of the femoral neck, the surgeon's experience is crucial to the procedure's success. This presents a significant challenge for less experienced surgeons, particularly in achieving accurate screw placement under fluoroscopic guidance, which may not only expose both the patient and surgical team to substantial radiation but also introduce serious complications associated with screw placement inaccuracies, including nonunion, fixation failure, and femoral head avascular necrosis (8, 9).

Moreover, these challenges are further exacerbated in specific populations. The femoral neck in Asian populations tends to be narrower, shorter, and exhibits a more varus neck-shaft angle compared to Western populations, increasing the difficulty of accurate screw placement and elevating the risk of cortical breach or fixation failure (10, 11). This underscores the need for precision technologies capable of adapting to patient-specific anatomy.

With advancements in imaging technology and computer science, the use of robotic systems such as the TiRobot (TINAVI Medical Technologies Co., Ltd., Beijing, China) in orthopedic surgery has become increasingly prevalent. The TiRobot integrates data processing, surgical planning, navigation, and precise positioning, enabling accurate screw placement during procedures (12). While the principle of precision alignment traditionally emphasized in joint arthroplasty, now it is gaining recognition in fracture fixation, particularly in anatomically complex regions like the femoral neck, where optimal screw trajectory and angulation must account for patient-specific morphology. In Asian populations, features such as narrower femoral necks and differing neck–shaft angles further influence alignment requirements. Robotic systems such as TiRobot assist surgeons in planning screw trajectories based on real-time imaging, improving the accuracy of placement and better accommodating regional anatomical characteristics.

Although several preliminary studies have explored the utilization of TiRobot-assisted surgery in the femoral neck fracture treatment, most remain limited in scope and lack comprehensive evaluation. In particular, there is a shortage of robust clinical data comparing TiRobot-assisted navigation with conventional freehand screw fixation in terms of cost-efficiency, safety, and functional outcomes. Therefore, this study aims to address this gap by offering insightful information on the clinical efficacy and cost-effectiveness of TiRobot-assisted fixation in femoral neck fractures through a comparative analysis, with a particular focus on surgical precision, cost-effectiveness, and the system's ability to accommodate anatomical variability through more accurate trajectory planning.

## Methods

### Study design

This retrospective cohort study included patients diagnosed with femoral neck fractures who had internal fixation surgery at Wuhan Union Hospital, a tertiary care center, between July 2023 and March 2024. Data were collected from April 2024 to July 2025. A total of 50 patients were enrolled and allocated into two groups based on the type of surgical intervention received: the TiRobot group (*n* = 25), treated with TiRobot-assisted cannulated screw fixation, and the traditional surgery group (*n* = 25), treated with conventional freehand cannulated screw fixation.

### Inclusion criteria

1. Age ≥ 18 years;
2. Underwent surgery as the initial treatment;
3. Diagnosed femoral neck fracture (displaced or non-displaced) treated with either TiRobot-assisted or traditional freehand cannulated screw fixation during the study period;
4. Complete follow-up data is available for a minimum of 12 months.

### Exclusion criteria

1. Evidence of femoral head necrosis on the affected hip before surgery;
2. Incomplete medical records or insufficient follow-up data;
3. Inability to bear surgery because of comorbidities such as severe hepatic, renal, or cardiovascular diseases;
4. Severe comorbidities that could affect long-term follow-up or evaluation of the study outcomes.

### Preoperative preparation

All patients enrolled in the study underwent a comprehensive preoperative evaluation. For those with chronic medical conditions, a multidisciplinary assessment was conducted by anesthesiologists, respiratory physicians, and cardiologists to evaluate surgical risk and manage comorbidities. Medical complications were actively treated to eliminate potential contraindications and ensure that patients were optimized for surgery. Prior to the initiation of the robotic procedure, all components of the TiRobot system were thoroughly inspected. Standard preoperative preparations were conducted for the robotic arm, optical tracking system, workstation, and C-arm x-ray machine. Power connections were verified, equipment functionality was confirmed, and the system was powered on. The surgical team logged into the TiRobot platform, entered relevant patient information, and selected the appropriate surgical tools within the system in preparation for intraoperative navigation and robotic guidance.

### Surgical technique

Traditional surgery group: patients in this group underwent percutaneous cannulated screw internal fixation using a conventional freehand technique. Each patient was given general anesthesia and put in a supine position. Following anatomical reduction of the femoral neck fracture under C-arm fluoroscopic guidance, three Kirschner wires were placed for temporary fixation. Intraoperative fluoroscopy was utilized to assess the anteroposterior (AP) and lateral view of the hip and verify the position and depth of the Kirschner wires. If malpositioning was detected, the wires were removed and repositioned. The tip of each wire was positioned approximately 0.5 mm below the femoral head cartilage. Once satisfactory alignment was achieved, the length of each Kirschner wire was measured, and cannulated screws were sequentially inserted along their respective trajectories. Final fluoroscopy was performed to confirm correct screw placement, after which the surgical wound was closed with sutures.

**Figure 1:**
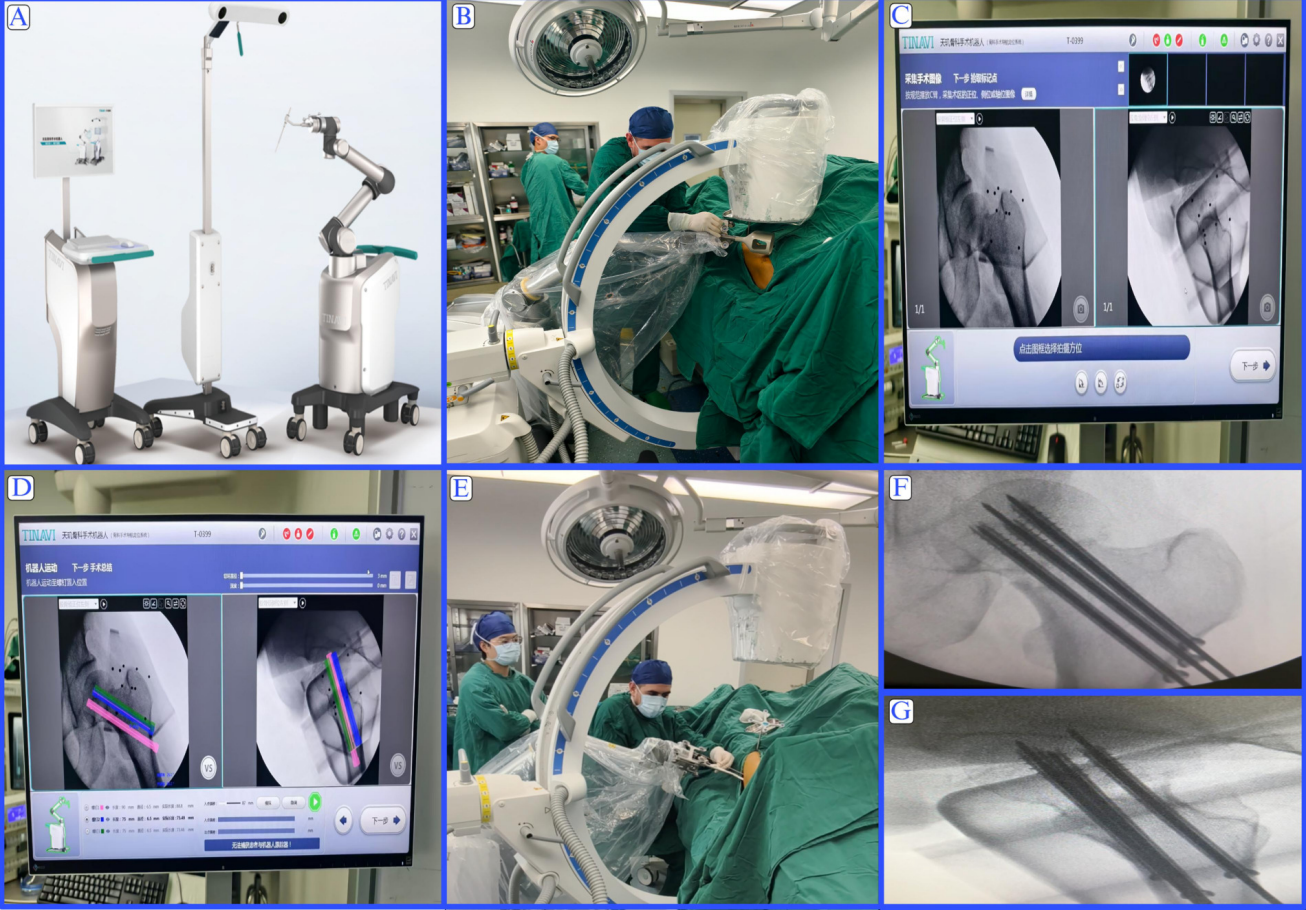
**(A)** The TiRobot surgical robotic system, consisting of a planning and control unit (left), optical tracking device (center), and robotic arm (right). **(B)** Intraoperative setup showing the patient positioned supine on an orthopedic traction table under general anesthesia, with C-arm fluoroscopy used to confirm fracture reduction and establish the spatial coordinate system for navigation. **(C)** Fluoroscopic interface on the TiRobot console displaying anteroposterior (AP) and lateral images for system calibration and verification. **(D)** Virtual screw trajectory planning on the TiRobot software interface. The surgeon defines optimal entry points and trajectories, shown as color-coded lines according to the patient's anatomy. **(E)** The TiRobot arm positions the guiding sleeve at the calculated entry point according to the preplanned trajectory. Real-time optical tracking feedback ensures submillimetric spatial accuracy during robotic movement, while the surgeon performs manual guidewire drilling through the sleeve. **(F,G)** Intraoperative fluoroscopic confirmation of accurate and parallel placement of three cannulated screws within the femoral neck and head.

TiRobot group: patients were positioned supine on the orthopedic traction table with the afflicted limb being subjected to consistent traction under general anesthesia. AP and lateral C-arm fluoroscopy was used to evaluate fracture reduction. Upon achieving satisfactory reduction, the robot was positioned and draped with a sterile plastic cover. A tracker was mounted on the robotic arm, and another tracker was placed on the ipsilateral anterior superior iliac spine. All ten calibration points of the trackers were confirmed to be visible on both lateral and AP fluoroscopic images. The x-rays were then forwarded to the robotic system. Using the software interface, the surgeon planned the trajectory for three screws depending on the patient's anatomy and fracture characteristics. The system immediately calculated the appropriate screw length and directed the robotic arm to the planned entry points. A drill sleeve was mounted and aligned in the positioning guide, through which guiding wires were placed into the femoral neck. After confirming the correct placement of the guiding wires via fluoroscopy, three cannulated screws were inserted. Final fluoroscopic verification was performed, followed by wound closure. The overall workflow of the TiRobot-assisted femoral neck screw fixation procedure is illustrated in Figure 1.

### Surgical team and experience

All procedures in both the TiRobot and traditional surgery groups were performed by senior orthopedic trauma surgeons with more than 10 years of experience in hip fracture surgery and proficiency in percutaneous cannulated screw fixation. The same surgical team performed both robotic and freehand procedures. Prior to the study period, the surgeons had completed the required institutional training and certification for TiRobot-assisted procedures, ensuring familiarity with the system and minimizing the influence of a learning curve on operative performance.

### Postoperative management and follow up

All patients received standardized postoperative care, including prophylactic antibiotics, thromboprophylaxis, pain management, and rehabilitation guidance. On the first postoperative day, AP and lateral hip radiographs were taken to assess implant positioning and fracture alignment. Patients were instructed to avoid excessive adduction, abduction, and external rotation of the injured hip during the first six weeks after surgery to promote optimal healing.

Early rehabilitation was initiated under the guidance of a physiotherapist. Isometric quadriceps contractions, ankle pump exercises, and gentle range-of-motion activities for the knee and hip were encouraged from the first postoperative day to maintain muscle tone and prevent venous thromboembolism. Partial weight-bearing with the aid of crutches or a walker was typically permitted at 6–8 weeks postoperatively, depending on radiographic evidence of early callus formation and fracture stability. Progressive weight-bearing was gradually introduced between 8 and 12 weeks, with full weight-bearing generally allowed at 12–16 weeks once radiographs confirmed sufficient bone healing. Strengthening and gait training exercises were advanced accordingly to restore mobility, balance, and hip function.

**Figure 2:**
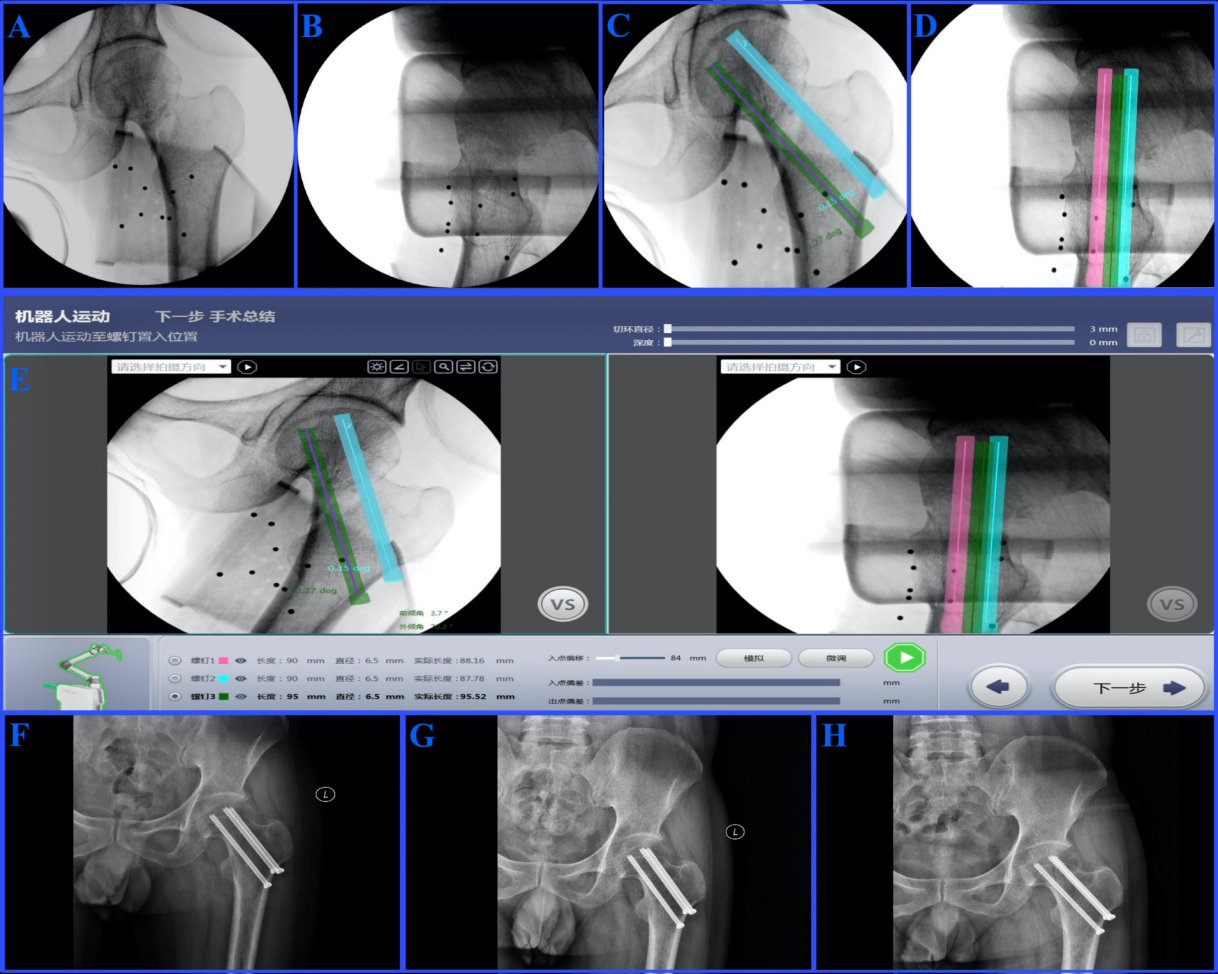
**(A,B)** Anteroposterior (AP) and lateral x-rays of the fractured femoral neck prior to fixation; **(C,D)** Planned trajectories for screw insertion displayed on the TiRobot software interface in AP and lateral; **(E)** AP and lateral views in the software interface showing the calculated screw lengths and alignment for precise fixation; **(F)** AP x-ray on postoperative day 1 demonstrating proper screw placement and satisfactory fracture reduction; **(G)** AP x-ray at 12 months postoperative, showing fracture healing and screw positioning; **(H)** AP x-ray at 20 months postoperative, confirming stable fixation without complications.

Postoperative follow-up was conducted over a period of 12 to 20 months. During the first six months, patients were followed up monthly; between six and twelve months, follow-up visits occurred at least once every three months; and after the first year, follow-up was conducted at least once every six months (Figure 2).

### Data collection

Following application of strict exclusion and inclusion criteria, data were extracted from the medical records of the hospital, imaging archives, operative documentation and follow up records. Only patients with complete clinical, surgical, and radiographic data were included. The data collection process was conducted between March and mid-April 2025. Data extraction was initially performed by one researcher and subsequently verified by a second independently trained reviewer to ensure data accuracy and quality control.

During the study period, all patients who met the eligibility criteria and underwent TiRobot-assisted cannulated screw fixation were included consecutively in the TiRobot group (*n* = 25). The decision to use robotic assistance was based solely on system availability and patient willingness, and not by surgeon preference, fracture characteristics, or patient demographics. Given that Wuhan Union Hospital is one of the top ten medical centers in China and manages a high patient volume, a computer-assisted matched sampling method was employed to establish a comparable traditional surgery group. Specifically, 25 patients were retrospectively selected from a pool of patients who met the same eligibility criteria and underwent conventional freehand percutaneous cannulated screw fixation during the same timeframe.

Selection of the traditional surgery cohort was performed using a computer-assisted 1:1 matching protocol to minimize selection bias. Matching was conducted based on clinically relevant variables known to influence fracture characteristics and surgical outcomes. Exact matching was applied for Garden classification, Pauwels classification, sex, and mechanism of injury, given their importance in determining fracture morphology and fixation strategy. For continuous variables, age (±5 years), BMI (±2 kg/m²), and time from injury to surgery (±1 day) were matched using predefined tolerance ranges to ensure close comparability. This sampling strategy ensured group comparability and minimized potential selection bias.

Preoperative AP and lateral hip radiographs were independently evaluated by two experienced orthopedic surgeons using the Garden system for displacement (13) and the Pauwels system for fracture inclination (14). In cases where classification differed between the two reviewers, a third senior consultant orthopedic surgeon provided the final determination to ensure classification accuracy and consistency. The same methodology was applied to assess postoperative and follow-up radiographs, using standardized criteria to evaluate screw positioning, fracture alignment, and healing progression. As the majority of patients had Garden type IV or III fractures and Pauwels type III or II fractures, the accuracy of fracture reduction was evaluated using the Haidukewych criteria (15), with reductions graded as: poor (displacement >5 mm, angulation >10°), fair (displacement 4–5 mm, angulation <10°), good (displacement 2–3 mm, angulation <5°), or excellent (displacement <2 mm, no angulation). Screw placement accuracy was assessed using a previously established model (16), where a line was drawn through the central axis of the three screws, and the angles between screw pairs were measured. The total of these angles represented screw parallelism.

Surgical safety was evaluated by recording intraoperative parameters and postoperative complications. Economic efficiency was assessed by directly comparing surgical costs between the two groups and indirectly through analysis of the time from surgery to discharge and the total hospitalization duration. The Harris hip score (17), which assesses function, pain, deformity, and range of motion, was used to evaluate functional recovery at the 12-month follow-up. Scores were categorized as poor (<70), fair (70–79), good (80–89), or excellent (90–100).

### Statistical analysis

Statistical analysis was conducted using SPSS software, version 26.0 (IBM Corp., Armonk, NY, USA). The mean ± standard deviation (SD) was utilized for continuous variables with a normal distribution, while the median with interquartile range (IQR) was used to represent data that was not normally distributed. Categorical variables were summarized as percentages and frequencies. Group comparisons were carried out using the Mann–Whitney *U*-test or independent samples *t*-test for continuous variables, based on the distribution, and the chi-square test or Fisher's exact test for categorical variables.

As this was a retrospective cohort study, no formal a-priori sample size calculation was performed. A *post-hoc* power analysis was performed using anteroposterior screw parallelism as the primary continuous outcome. The mean AP parallelism was 0.90 ± 0.48° in the TiRobot group and 1.47 ± 0.91° in the traditional group, yielding a pooled SD of 0.73° and a standardized effect size (Cohen's d) of 0.78. Using a two-sided independent-samples t-test with α = 0.05 and *n* = 25 patients per group (G*Power 3.1), the achieved power (1−β) was 0.792 (79.2%). This indicates that the present sample size is close to the conventional 80% power threshold for this primary outcome.

## Results

### Patients’ characteristics

A total of 50 patients were allocated equally to two groups: the TiRobot group (*n* = 25) and the traditional surgery group (*n* = 25). As presented in Table 1, no significant differences were observed in age, sex, BMI, comorbidities, cause or side of injury, Garden classification, Pauwels classification, time from injury to surgery, or follow-up duration (all *p* > 0.05).

**Table 1:** Comparison of patients characteristics between the two groups.

Patients’ characteristics	TiRobot group (*n* = 25)	Traditional surgery group (*n* = 25)	*p*-value
Age (years, median ± IQR)	52 ± 19	55 ± 21.5	0.382*
Gender (*n*, %)			0.571***
Male	14 (56%)	12 (48%)	
Female	11 (44%)	13 (52%)	
BMI (mean ± SD)	21.87 ± 3.04	21.81 ± 2.55	0.935**
Comorbidities (*n*)			0.581****
None	18 (72%)	14 (56%)	
HTN	3 (12%)	3 (12%)	
DM	1 (4%)	1 (4%)	
Osteoporosis	3 (12%)	7 (28%)	
Causes of injury (*n*)			0.667****
Fall	21 (84%)	23 (92%)	
Traffic accidents	3 (12%)	2 (8%)	
Crush	1 (4%)	0 (0%)	
Injury side (*n*)			0.396***
Right	11 (44%)	15 (60%)	
Left	14 (56%)	10 (40%)	
Garden classification (*n*)^†^			>0.99****
II	3 (12%)	4 (16%)	
III	9 (36%)	8 (32%)	
IV	13 (52%)	13 (52%)	
Pauwels classification			0.905****
I	2 (8%)	2 (8%)	
II	9 (36%)	7 (28%)	
III	14 (56%)	16 (64%)	
Time from injury to operation (days, mean ± SD)	2.90 ± 1	2.83 ± 0.93	0.802**
Follow-up period (months, median ± IQR)	14.4 ± 3.1	15.47 ± 2.61	0.121*

^†^ Garden I fractures were not observed in either group.

“*Mann–Whitney *U*-test; **Independent samples *t*-test; ***Chi-square test; ****Fisher's exact test”.

BMI, body mass index; DM, diabetes mellitus; HTN, hypertension; IQR, interquartile range; SD, standard deviation.

### Surgical operation indexes

Compared with traditional surgery, the TiRobot group had a reduced operative duration (*p* = 0.028), less intraoperative blood loss (*p* < 0.001), and fewer fluoroscopy exposures (*p* < 0.001). Screw parallelism in both anteroposterior and lateral views was significantly better in the TiRobot group (*p* = 0.008 and *p* = 0.010, respectively). Fracture reduction, as assessed by the Haidukewych criteria, was comparable between the groups (*p* = 0.702), as shown in Table 2.

**Table 2:** Comparison of surgical operation indexes between the two groups.

Characteristics	TiRobot group (*n* = 25)	Traditional surgery group (*n* = 25)	*p*-value
Operation duration (min, median ± IQR)	68 ± 14	81 ± 23.5	0.028*
Blood loss (mL, mean ± SD)	27.68 ± 12.99	49.80 ± 15.03	<0.001**
Fluoroscopy frequency (times, mean ± SD)	12.24 ± 1.94	20.24 ± 2.47	<0.001**
Anteroposterior screw parallelism (degree, mean ± SD)	0.90 ± 0.48	1.47 ± 0.91	0.008**
Lateral screw parallelism (degree, mean ± SD)	1.42 ± 0.62	2.03 ± 0.94	0.010**
Haidukewych criteria^†^ (*n*, %)			0.702***
Excellent	22 (88%)	20 (80%)	
Good	3 (12%)	5 (20%)	

^†^ No patients were rated as fair or poor in either group based on Haidukewych criteria.

“*Mann–Whitney *U*-test; **Independent samples *t*-test; ***Fisher's exact test”.

### Hip function and complications

Table 3 shows that all patients achieved fracture healing. The TiRobot group had a significantly higher Harris hip score (*p* = 0.014) and a greater proportion of excellent outcomes. Screw loosening occurred only in the traditional group, with no other complications reported in either group.

**Table 3:** Comparison of clinical outcomes and postoperative complications between the two groups.

Outcome/complication	TiRobot group (*n* = 25)	Traditional surgery group (*n* = 25)	*p*-value
Fracture healed cases (*n*, %)	25 (100%)	25 (100%)	
Harris hip score (points, median ± IQR)	94 ± 8.5	87 ± 13.5	0.014*
Excellent (*n*, %)	17 (68%)	9 (36%)	
Good (*n*, %)	5 (20%)	8 (32%)	
Fair (*n*, %)	3 (12%)	7 (28%)	
Poor (*n*, %)	0 (0%)	1 (4%)	
Complications (*n*, %)			
Screw loosening	0 (0%)	2 (8%)	0.245**
Other complications^†^	0 (0%)	0 (0%)	

^†^ Other complications: surgical site infection, internal fixation failure, fracture redisplacement, and femoral head necrosis.

“*Mann–Whitney *U*-test; **Fisher's exact test”.

### Cost-effectiveness

Patients in the TiRobot group had a shorter time to discharge and total hospitalization duration (*p* < 0.001 and *p* = 0.039, respectively). However, the surgical cost was significantly higher in the TiRobot group (*p* < 0.001), as shown in Table 4.

**Table 4:** Comparison of hospitalization duration and cost-effectiveness between the two groups.

Variable	TiRobot group (*n* = 25)	Traditional surgery group (*n* = 25)	*p*-value
Time from surgery to discharge (days, mean ± SD)	5.01 ± 1.46	7.03 ± 1.86	<0.001**
Hospitalization days (median ± IQR)	7 ± 3	8 ± 3	0.039*
Total surgery cost (CNY, mean ± SD)	28,998.31 ± 4,659.88	17,653.63 ± 4,461.55	<0.001**

“*Mann–Whitney *U*-test; **Independent samples *t*-test”.

## Discussion

With the rapid evolution of medical technologies and the continuous refinement of orthopedic robotic systems both domestically and internationally, robotic-assisted platforms have seen growing integration into clinical orthopedic practice (18), driven by the demand for high-precision, reproducible outcomes and the shift toward approaches that better account for anatomical variability. This study offers a robust comparative assessment of TiRobot-assisted vs. freehand cannulated screw fixation for femoral neck fractures. The findings underscore the clinical advantages of robotic assistance, particularly in terms of surgical precision, intraoperative safety, and functional recovery. These advantages are especially pronounced in anatomically demanding cases, such as those seen in Asian populations, where narrower and more varus-aligned femoral necks pose greater technical challenges to freehand fixation. At the same time, the study thoughtfully addresses the economic implications of robotic surgery, presenting a balanced perspective on the trade-offs between improved perioperative outcomes and increased procedural costs. These results contribute valuable real-world evidence to the ongoing discourse on the clinical utility and cost-effectiveness of robotic technologies in orthopedic trauma care, and support the shift toward more precise, imaging-guided alignment strategies in fracture management.

In this study, the demographic characteristics and baseline clinical parameters were well-matched between the TiRobot and traditional surgery groups, thereby minimizing potential confounding and strengthening the validity of intergroup comparisons. Notably, the majority of patients in both groups presented with Garden type IV or III and Pauwels type III or II femoral neck fractures, which differs from previous studies (18–20) that often included a higher prevalence of less severe fracture types. This discrepancy may be attributed to referral patterns within China's tiered healthcare system. As Wuhan Union Hospital is among the top ten tertiary medical centers in the country, it typically receives referrals for complex and advanced cases, while less severe fractures are frequently managed at lower-tier regional hospitals. This context underscores the relevance and applicability of the study's findings to higher-complexity clinical scenarios.

Consistent with previous studies (18, 21, 22), the TiRobot group demonstrated a significantly shorter operative duration than the traditional group (*p* = 0.028). However, only one earlier study (23) published in 2019 indicated no substantial difference in operative time between the two groups. This is likely due to limited surgeon familiarity with the robotic system at the time in which the system was first introduced. In addition to reduced operative time, our findings demonstrated significantly lower intraoperative blood loss in the TiRobot group (*p* < 0.001), aligning with the findings from several studies (18, 19, 23). In contrast, a study by Liu et al. (22) did not observe a statistically significant variance, this discrepancy may be explained by differences in fracture severity. Our finding may be influenced by the predominance of Garden III–IV and Pauwels II–III fractures in this study; in such cases, open reduction is more frequently required when closed reduction fails, inherently leading to greater bleeding risk, particularly in the traditional surgery group.

In terms of radiation exposure, this study showed a significantly lower fluoroscopy frequency in the TiRobot group (*p* < 0.001), corroborating the findings of previous studies (18, 22, 23). However, one study (19) did not observe a statistically significant difference, possibly due to variability in imaging protocols or surgical experience. While both groups achieved comparable fracture reduction quality as assessed by the Haidukewych criteria (*p* = 0.702), the TiRobot group exhibited significantly better screw parallelism in both anteroposterior and lateral views (*p* = 0.008 and *p* = 0.010, respectively) aligning with previous findings (19) suggesting improved mechanical stability. This enhanced accuracy is likely attributable to the robotic system's integration of real-time imaging and millimeter-level precision, which may be particularly beneficial in improving consistency and outcomes, especially for surgeons with less operative experience.

Functionally, the TiRobot group demonstrated significantly better Harris hip scores at 12-month follow-up (*p* = 0.014), consistent with previous studies (18, 22). Both groups achieved a 100% fracture healing rate without major complications. However, two cases of screw loosening were noted in the traditional group, while no such complications were observed in the TiRobot cohort, further supporting the potential for enhanced fixation stability through robotic precision.

From an economic perspective, while the TiRobot-assisted approach was associated with a significantly higher surgical cost (*p* < 0.001), it also led to earlier patient discharge (*p* < 0.001) and reduced overall hospital stay (*p* = 0.039), indicating potential perioperative cost savings. This is the only study that we are aware of that thoroughly assesses the cost-effectiveness of TiRobot-assisted surgery in femoral neck fractures. While one previous study (19) reported surgical cost and hospitalization duration, it was limited to patients without comorbidities, thereby reducing its generalizability. These findings suggest that improved perioperative efficiency may partially offset the increased procedural costs. However, the current high cost of robotic surgery in China and its lack of coverage by Chinese national medical insurance present practical barriers to widespread adoption.

This study has several limitations. First, its single-center retrospective design inherently carries a risk of selection bias and limits the findings’ applicability to other healthcare settings with differing surgical expertise, patient populations, or institutional protocols. Although TiRobot-assisted cases were enrolled consecutively, the allocation of patients was partly influenced by robotic system availability during the study period, and therefore unmeasured confounding cannot be fully excluded.

Second, the statistical power of our study is constrained by the sample size (*n* = 50). While a *post-hoc* power analysis indicated that the study had approximately 79.2% power to detect the observed difference in screw parallelism, the study is underpowered to draw definitive conclusions, particularly regarding low-frequency but clinically important complications such as screw loosening, fixation failure, or avascular necrosis. Consequently, these complication-related findings should be interpreted as exploratory and hypothesis-generating rather than confirmatory.

Third, although the follow-up period extended up to 20 months, this duration may be inadequate to fully assess long-term complications such as avascular necrosis, late-onset hardware failure, or functional decline. Additionally, while all procedures were performed by experienced surgeons who had completed a standardized training protocol for the TiRobot system, a minimal learning curve effect for the TiRobot system cannot be entirely excluded. Future studies should prioritize prospective, multi-center randomized controlled designs with larger patient populations and extended follow-up durations to validate and expand upon these findings.

## Conclusion

This study provides compelling evidence that TiRobot-assisted cannulated screw fixation offers several clinical advantages over traditional freehand techniques in the femoral neck fracture treatment. Robotic assistance improves surgical accuracy, minimizes intraoperative blood loss, and reduces fluoroscopy exposure. These advantages translate into better functional recovery, earlier discharge, and shorter overall hospitalization. Although the total surgical costs were higher for robotic-assisted procedures, the enhanced perioperative efficiency and improved clinical outcomes suggest that TiRobot assistance offers a favorable cost-benefit profile for managing femoral neck fractures. These findings support the role of robotic assistance in enabling surgeon-driven, anatomy-guided precision alignment in femoral neck fracture fixation, particularly for populations with complex bony morphology, such as those in Asia.

## Data Availability

The datasets presented in this article are not readily available because of confidentiality but are available from the corresponding author on reasonable request. Requests to access the datasets should be directed to liuguohui@hust.edu.cn.
